# NAFLD and cardiovascular diseases: a clinical review

**DOI:** 10.1007/s00392-020-01709-7

**Published:** 2020-07-21

**Authors:** Philipp Kasper, Anna Martin, Sonja Lang, Fabian Kütting, Tobias Goeser, Münevver Demir, Hans-Michael Steffen

**Affiliations:** 1 Department of Gastroenterology and Hepatology, Faculty of Medicine, and University Hospital Cologne, University of Cologne, Kerpener Str. 62, 50937 Cologne, Germany; 2 Department of Medicine, University of California, La Jolla, San Diego, USA; 3 Department of Hepatology and Gastroenterology, Charité University Medicine, Campus Virchow Clinic, Berlin, Germany; 4 Hypertension Center, Faculty of Medicine, and University Hospital Cologne, University of Cologne, Cologne, Germany

**Keywords:** Non-alcoholic fatty liver disease, NAFLD, Cardiovascular disease, Metabolic syndrome, Diabetes, Insulin resistance, Hyperinsulinemia, Systemic inflammation

## Abstract

Non-alcoholic fatty liver DISEASE (NAFLD) is the most common chronic liver disease in Western countries and affects approximately 25% of the adult population. Since NAFLD is frequently associated with further metabolic comorbidities such as obesity, type 2 diabetes mellitus, or dyslipidemia, it is generally considered as the hepatic manifestation of the metabolic syndrome. In addition to its potential to cause liver-related morbidity and mortality, NAFLD is also associated with subclinical and clinical cardiovascular disease (CVD). Growing evidence indicates that patients with NAFLD are at substantial risk for the development of hypertension, coronary heart disease, cardiomyopathy, and cardiac arrhythmias, which clinically result in increased cardiovascular morbidity and mortality. The natural history of NAFLD is variable and the vast majority of patients will not progress from simple steatosis to fibrosis and end stage liver disease. However, patients with progressive forms of NAFLD, including non-alcoholic steatohepatitis (NASH) and/or advanced fibrosis, as well as NAFLD patients with concomitant types 2 diabetes are at highest risk for CVD. This review describes the underlying pathophysiological mechanisms linking NAFLD and CVD, discusses the role of NAFLD as a metabolic dysfunction associated cardiovascular risk factor, and focuses on common cardiovascular manifestations in NAFLD patients.

## Introduction

Non-alcoholic fatty liver disease (NAFLD) has become the most common chronic liver disease in Western countries with a constantly increasing incidence and prevalence, causing a tremendous clinical and economic burden [1–3].

In the majority of patients, NAFLD is associated with metabolic comorbidities such as obesity, type 2 diabetes mellitus (T2DM), or dyslipidemia, and is generally considered as the hepatic manifestation of the metabolic syndrome [4].

The diagnosis of NAFLD requires evidence of hepatic steatosis, by either imaging or histology, and the exclusion of secondary causes of hepatic fat accumulation, such as use of steatogenic medication (e.g. corticosteroids, amiodarone, methotrexate), hereditary disorders (e.g. Wilson’s disease, alpha-1 antitrypsin deficiency) or viral infections (e.g. hepatitis C infection). Additionally, daily alcohol consumption must not exceed 30 g for men and 20 g for women per day [5]. However, these alcohol consumption thresholds are not unequivocal as lower limits of 20 g/day and 10 g/day, respectively, have been advocated by guidelines from Germany and the Asian-Pacific region [2, 6].

Histologically, NAFLD can be categorized into non-alcoholic fatty liver (NAFL) and non-alcoholic steatohepatitis (NASH). While NAFL is defined as the presence of ≥ 5% hepatic steatosis without evidence of hepatocyte injury, NASH is defined as the presence of hepatic steatosis and accompanying lobular inflammation with hepatocyte injury (e.g. hepatocyte ballooning), with or without fibrosis [7, 8].

The natural history of disease progression in NAFLD is highly variable, e.g. in the placebo group of the CENTAUR study (cenicriviroc, a dual CCR2/CCR5 antagonist) the fibrosis response worsened for those who initially improved between the first and second year and vice versa for those who initially worsened after the first year [9]. While most patients have mild steatosis, approximately 20–30% of cases develop NASH with progressive fibrosis, and of those, approximately 20% will progress to cirrhosis with an increased risk of hepatocellular carcinoma [10, 11]. Risk factors associated with NAFLD progression include genetic alterations, increasing age, inflammation, gut dysbiosis and metabolic abnormalities such as insulin resistance and hyperinsulinemia [10–13].

The global prevalence of NAFLD has been estimated to be approximately 25%, while the global prevalence of NASH ranges from around 3 to 5% [14]. Across European countries NAFLD affects approximately 1 in 4 members of the general population with a gradient of higher prevalence from Southern to Northern Europe [15].

The mortality rate among NAFLD patients is substantially higher than in the general population [16–18]. NAFLD patients with advanced fibrosis or cirrhosis (≥ fibrosis stage F3) have the highest risk of liver-related death, while cardiovascular events and non-hepatic malignancies are the most common complications in patients with early-stage NAFLD (< F3) [16, 18–24].

The majority of NAFLD patients are affected from early NAFLD stages and are often characterized by additional cardiometabolic risk factors [25]. The identification of these patients has the potential to detect subjects at high cardiometabolic risk who are candidates for therapeutic interventions aimed at prevention of progressive NAFLD as well as atherosclerotic cardiovascular disease (CVD) [26, 27].

This review analyzes the relationship between NAFLD and CVD, describes the underlying pathophysiological mechanisms that might link NAFLD to CVD and focuses on common cardiovascular manifestations in NAFLD patients.

## Pathophysiological mechanisms linking NAFLD and CVD

**Fig. 1:**
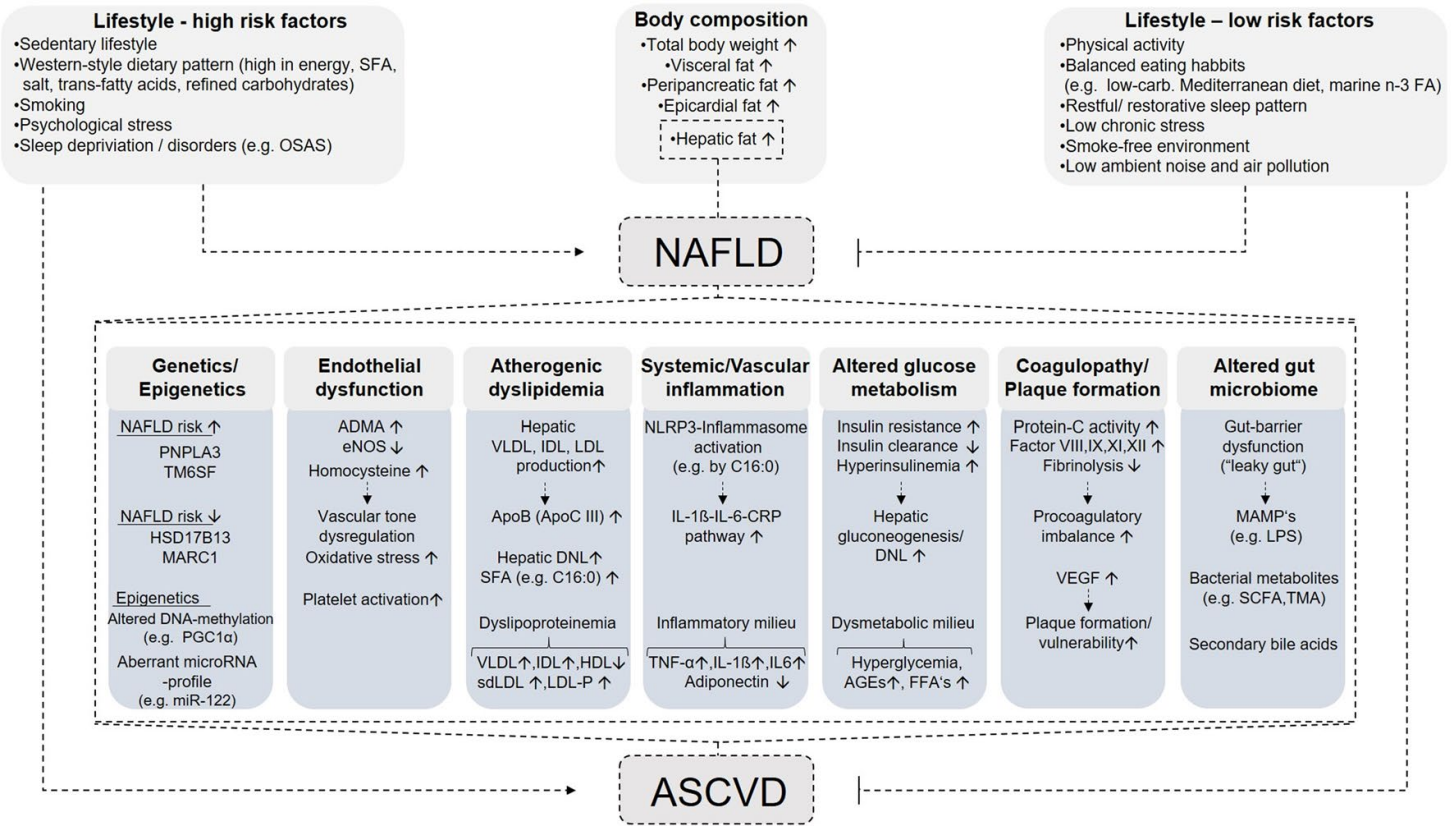
Pathophysiological mechanisms linking NAFLD and CVD (modified from: [12, 28, 29]). *SFA* saturated fatty acids, *OSAS* obstructive sleep apnea syndrome, *carb*. carbohydrates, *n*-*3 FA* omega-3 fatty acids, *NAFLD* non-alcoholic fatty liver disease; *PNPLA3* patatin-like phospholipase domain-containing protein 3, *TM6SF* transmembrane 6 superfamily 2 human gene, *HSD17B13* hydroxysteroid dehydrogenase 17beta 13, *MARC1* mitochondrial amidoxime reducing component 1, *PGC1α* peroxisome proliferator-activated receptor gamma coactivator 1-alpha, *miR* micro-RNA, *ADMA* asymmetric dimethylarginine, *eNOS* endothelial nitric oxide synthase, *VLDL* very-low-density lipoprotein, *IDL* intermediate-density lipoprotein, *LDL* low-density lipoprotein, *ApoB* apolipoprotein B, *ApoC III* apolipoprotein C3, *DNL* de novo lipogenesis, *HDL*-*C* high-density lipoprotein cholesterol, *sdLDL* small dense low-density lipoprotein, *LDL*-*P* low-density lipoprotein particles, *C16:0* palmitic acid, *IL*-*1β* interleukin-1β, *IL*-*6* interleukin-6, *CRP* C-reactive protein, *AGEs* advanced glycation end products, *FFAs* free fatty acids, *VEGF* vascular endothelial growth factor, *MAMPs* microbe-associated molecular pattern, *LPS* lipopolysaccharide, *SCFA* short-chain fatty acids, *TMA* trimethylamine; *ASCVD* atherosclerotic cardiovascular disease. Down arrows (↓) indicate decreased levels, and up arrows (↑) indicate increased levels

NAFLD and CVD are both manifestations of end-organ damage of the metabolic syndrome and a specific contribution of NAFLD to increased CVD risk is difficult to discern from the combination of these shared risk factors [28]. The underlying mechanisms linking NAFLD to CVD are very complex and simultaneously involve a number of different pathways [28, 29] (Fig. 1).

Dysfunctional visceral adipose tissue, as well as an increased accumulation of dysfunctional, ectopic fat in the liver and other organs such as the pericardium, pancreas, kidneys, or skeletal muscle, are closely related to adverse cardiometabolic outcomes [30]. This accumulation of (visceral and) ectopic fat and the subsequent release of fat-derived toxic metabolites together with an activation of inflammatory pathways instigates a cluster of local and systemic pathophysiological changes that ultimately leads to the development of both NAFLD and cardiovascular diseases, possibly via mechanisms beyond overweight and obesity [31, 32].

In NAFLD, hepatic fat accumulation results from an imbalance between lipid acquisition and lipid disposal mediated by the following pathways: inadaquate uptake of circulating lipids, increased hepatic de-novo lipogenesis (DNL), insufficient enhancement of compensatory fatty acid oxidation, and altered export of lipids as components of very low-density lipoproteins (VLDL) [33]. In detail, elevated lipid uptake and increased rates of DNL in NAFLD lead to an increased hepatic triglyceride accumulation with concomitant overproduction and secretion of large, triglyceride-enriched VLDL particles, which serve to mobilize liver fat for transport to peripheral tissues [12, 34]. In NAFLD, this overproduction of VLDL particles initiates a cluster of plasma lipoprotein abnormalities and an atherogenic dyslipidemia, which is characterized by high serum triglycerides and low high-density lipoprotein (HDL) cholesterol levels, as well as an atherogenic lipoprotein phenotype, including a predominance of small dense low-density lipoprotein (LDL) particles, and an accumulation of triglyceride-rich lipoproteins and their remnants, intermediate-density lipoprotein (IDL) [12, 34, 35]. Those apolipoprotein-B containing lipoproteins, in turn, are mainly involved in the development of atherosclerosis [12]. After penetration, accumulation and modification (oxidation) within the subendothelial vascular wall, apolipoprotein-B-containing lipoproteins serve as damage-associated molecular patterns (DAMPs) activating Toll-like receptors (TLRs) [12, 36]. TLR are components of the innate immune system, tasked with sensing invading pathogens or endogenous damage signals and initiating suitable immune response [37].

In this context, particularly the triglyceride-rich lipoproteins containing apolipoprotein C3 (ApoC3) play an important role, which are capable of activating the TLRs 2 and 4 via dimerization and thereby activating the so-called NLRP3 (NOD-like receptor family, pyrin domain-containing protein 3) inflammasome [12, 36]. Once activated by DAMPs, pathogen-associated molecular patterns (PAMPs) or other atherosclerosis-related stimuli, the NLRP3 inflammasome regulates the activity of one of its constituent proteins, the enzyme caspase-1, also known as IL (interleukin)-1β converting enzyme [38]. Activation of caspase-1 via the NLRP3 inflammasome leads to a proteolytic activation of proinflammatory cytokines of the IL-1β family and the subsequent induction of the IL-1 to IL-6 to CRP (C-reactive protein) inflammatory pathway, which is involved in the development of vascular inflammation and atherosclerotic cardiovascular disease [38]. The activation of TLRs by ApoC3 and the following activation of the NLRP3 inflammasome thereby provides an important link between atherogenic lipoprotein patterns, commonly observed in patients with NAFLD and vascular immune activation/inflammation [12]. An increased hepatic DNL in NAFLD is also associated with an increased hepatic palmitic acid (C 16:0) flux and an enrichment of palmitic acid in VLDL particles [12, 39]. Saturated fatty acids, such as palmitic acid, in turn, may also induce vascular inflammation by inducing dimerization and activation of TLRs 2 and 4, representing another mechanism by which NAFLD promotes the development of vascular damage and atherosclerosis [12, 40]. Higher palmitic acid or palmitoleic acid (16:1n-7) were associated with higher risks of all cause and cardiovascular mortality [41]. Following step-wise increments in dietary carbohydrate intake levels of both fatty acids rise and palmitoleic acid serves as a biomarker for an increased hepatic DNL [42, 43].

Abnormal glucose metabolism and hepatic insulin resistance are further major hallmarks of NAFLD, and crucial in both NAFLD and CVD pathogenesis [28, 29]. Disorders of glucose metabolism in NAFLD patients can be attributed to the underlying systemic inflammation, visceral obesity in conjunction with commonly increased body weight, but also to an increased accumulation of dysfunctional ectopic fatty tissue [12, 44]. In addition to an increased liver fat content, accumulation of pancreatic ectopic dysfunctional adipose tissue plays an important role in this context, which is essentially associated with insulin resistance and beta cell dysfunction [44–46]. Insulin resistance is accompanied by a compensatory persistent hyperinsulinemia, that is of central importance for the induction and maintenance of an unfavorable metabolic milieu (e.g. increased free fatty acid and glucose levels), whereby the prevailing insulin resistance further worsens and subsequently promotes the development of cardiometabolic disorders [12]. In detail, hyperinsulinemia is associated with increased hepatic glucose production which results in increased plasma glucose levels and persistently increased insulin levels, representing a self-reinforcing cycle. This condition is deteriorated by the fact that NAFLD decreases hepatic insulin clearance. At the same time high insulin activates two transcription factors, named sterol regulatory element-binding protein 1c (SREBP-1c) and carbohydrate-responsive element binding protein (ChREBP), yielding greater expression of various lipogenic enzymes involved in DNL [13]. This results in further accumulation of hepatic fat and the production of saturated fatty acids, which, together with the increased blood glucose levels, maintains the disturbed metabolic environment.

Insulin resistance and impaired insulin signaling affects various processes that are associated with atherogenesis, enhanced progression of atherosclerotic lesions and plaque vulnerability. Persistent hyperglycemia and postprandial glucose spikes promote oxidative stress with concomitant activation of inflammatory signaling pathways, inflammasome activation, and vascular inflammation via advanced glycation end products (AEG), as well as dysregulation of lipoprotein metabolism or continuing ectopic fat accumulation [12, 45, 47]. Furthermore, insulin resistance is associated with dysregulated neurohumoral activation of the renin–angiotensin–aldosterone system, fibrinolytic dysfunction via increased plasminogen activator inhibitor-1 (PAI-1) levels, the development of cardiac autonomic neuropathy, which may promote the development of systolic and diastolic dysfunction or cardiac arrhythmias, and endothelial dysfunction [12, 47–50].

Endothelial dysfunction, in turn, is an early step in the pathogenesis of atherosclerosis and hence also crucial in CVD development [28, 29, 51]. Endothelial dysfunction is related to superoxide-related oxidative stress, lipoprotein (e.g. ApoC3)-mediated vascular inflammation and selective vascular insulin resistance, and is characterized by decreased bioavailability of the vascular protective vasodilatory molecule nitric oxide (NO) [12, 29, 36, 52]. Increased levels of asymmetric dimethyl arginine (ADMA), which represents an endogenous antagonist of nitric oxide synthase (NOs), result in reduced NO availability, that may lead to disturbed vasomotor regulation or vascular permeability and platelet dysfunction [28, 29]. Elevated ADMA levels originating, among other things, from impaired ADMA breakdown within the liver and can often be detected in patients with NAFLD [29]. Another factor involved in the development of endothelial dysfunction is hyperhomocysteinemia. Elevated serum levels of homocysteine induces oxidative stress by reduced repletion of glutathione stores, which is associated with impaired NO formation, increased vascular resistance, and enhanced platelet activation [29]. Plasma levels of homocysteine were increased in children and adults with NAFLD [53, 54], however, patients with steatohepatitis exhibited lower levels of homocysteine than subjects without [55]. Patients with NAFLD may also be at increased risk for the development of atherosclerotic CVD due to a procoagulant imbalance. They frequently have increased serum levels of the coagulation factors FVIII, FIX, FXI and FXII, which is accompanied by increased circulating concentrations of fibrinogen, von Willebrand factor and PAI-1, while antithrombin III and protein C are decreased [28, 29, 56]. An additional aspect that may influence atherogenesis and plaque instability in NAFLD patients includes altered serum concentrations of vascular endothelial growth factor (VEGF). Elevated serum levels of VEGF and signs of active angiogenesis, indicative of vascular remodelling, can be observed in NAFLD patients and are simultaneously associated with plaque formation and plaque instability [57]. However, the data available so far are inconsistent and the functional role of VEGF in atherogenesis of NAFLD patients still needs further investigations [29].

Besides fat accumulation in the liver, cardiac accumulation of ectopic fatty tissue, which includes the myocardial fat and the adipose tissue surrounding the heart, is a central aspect in the pathogenetic relationship between NAFLD and CVD [58]. The ectopic fat surrounding the heart can be classified into two different fat compartments separated by the pericardium: the (intra-) epicardial fat, located between the myocardium and the visceral serous pericardium, and the (extra-) pericardial fat, located externally to the fibrous pericardium [59, 60]. In particular the epicardial adipose tissue has unique properties. It plays a critical role in the activation of the cardiac nervous system and the thickness of epicardial adipose tissue is associated with the severity of obstructive sleep apnea syndrome as well as cardiovascular and liver damage in patients with NAFLD [61–64]. Due to its anatomical intimacy with the heart muscle, epicardial adipose tissue shares an unobstructed microcirculation with the adjacent myocardium, where the transit of both, hormonal factors and pluripotent cells, takes place [59]. Under healthy conditions and low oxidative stress the adipocytes of the pericardium nourish the adjacent myocardium (e.g. via fatty acid metabolism) and secrete protective substances, such as adiponectin, that mediate anti-inflammatory, antioxidative, anti-fibrotic, and anti-atherogenic cardioprotective effects [59, 60]. In a state of systemic inflammation and concomitant accumulation of the epicardial fat, induced by obesity, NAFLD, or other metabolic and dyslipidemic disorders, the biological characteristics of epicardial fat tissue changes [59, 60]. The release of adiponectin declines, whereas the epicardial fat depot synthesizes more proinflammatory cytokines (e.g. leptin, tumor necrosis factor-a, IL 1-b and IL-6, and resistin) that promote the infiltration of macrophages, destroy microvascular systems, and activate profibrotic pathways [59, 65, 66]. The induction of this inflammatory phenotype results in pathological changes of the coronary arteries embedded in this fatty tissue, and structural changes of the adjoining myocardium, such as inflammation and ventricular fibrosis, which can ultimately lead to the development and progression of coronary heart disease, atrial fibrillation and cardiac dysfunction (e.g. heart failure with preserved ejection fraction) [59, 60, 66]. Furthermore, it should be noted that the secretion of proinflammatory cytokines from epicardial fatty tissue into the general circulation reinforces the prevailing systemic proinflammatory milieu, which in turn further increases the extent of underlying (cardio-)metabolic disorders and promotes the deposition of further epicardial fat, resulting in a vicious circle [66]. Since NAFLD promotes a state of systemic inflammation, as mentioned before, and has been associated with an expansion of epicardial tissue, this likely represents another central pathophysiological link between NAFLD and CVD.

Genetic alterations involved in NAFLD pathobiology, include polymorphisms in the patatin-like phospholipase domain-containing 3 (PNPLA3) and the transmembrane 6 superfamily member 2 (TM6SF2) genes. Modifications of these genes confer an increased risk of developing fatty liver, NASH, fibrosis and hepatocellular carcinoma [27]. On the other hand, there are also protective genetic variants that decrease the risk for severe liver fibrosis or cirrhosis. A splice variant in HSD17B13 (encoding the hepatic lipid droplet protein hydroxysteroid 17-beta dehydrogenase 13) was associated with decreased serum aminotransferase levels as well as a reduced risk of NASH or fibrosis and also mitigated the risk of liver injury in persons who were genetically predisposed to fatty liver disease by the PNPLA3 (I148M) variant [67]. Recently a common missense variant (A165T) in a gene called MARC1 (mitochondrial amidoxime reducing component 1) was identified as a novel genetic determinant of all-cause cirrhosis that was also associated with lower liver fat content, liver enzymes levels, and lower total and LDL cholesterol levels [68]. The mechanism by which MARC1 may contribute to liver damage and cirrhosis remains to be elucidated. Epigenetic modifications such as an altered promoter methylation of the hepatic energy regulator PPARγ coactivator 1 alpha (PGC1α) or changes in the expression of the hepatic micro-RNAs (e.g. miR-122), are predicted to be further major determinants of NAFLD disease progression [69].

Interestingly, recent studies suggest that carriers of the described genetic variants of PNPLA3 and TM6SF2 tend to have an apparent cardioprotective phenotype [70–72]. Lauridsen et al. demonstrated that the risk of ischemic heart disease increased stepwise with increasing liver fat content, however, using a Mendelian randomization approach PNPLA3 polymorphism was not causally linked to ischemic heart disease [71]. Moreover, in a large exome-wide association study of plasma lipids of > 300,000 individuals by Liu et al., genetic variants of PNPLA3 and TM6SF2 were not only strongly associated with fatty liver and progression to NASH, cirrhosis, and hepatocellular carcinoma, but also with lower blood triglycerides, lower LDL-cholesterol concentrations, and protection from coronary artery disease [72]. MARC1 variants were also not associated with the risk of coronary artery disease suggesting that therapeutic MARC1 antagonism may be useful for the prevention and treatment of liver disease without increasing coronary risk. However, to what extent these genetic modifications also influence the development of other cardiovascular diseases and the cardiovascular event rate requires further clarification.

In addition to genetic alterations or changes in body composition alterations in the gut microbiota composition, referred to as dysbiosis, have been identified as a contributing factor for the development of cardiometabolic diseases such as atherosclerosis, hypertension, T2DM or NAFLD [73, 74]. Among these diseases, there is a considerable overlap of gut bacterial taxa considered as beneficial or detrimental and despite repeatedly reproduced abundance patterns of specific bacteria, the heterogeneous study results did not reveal a consistent NAFLD specific gut microbiota signature [75, 76]. A common feature associated with dysbiosis is an impaired intestinal barrier function with subsequent increased mucosal barrier permeability. Consequently intestinal microbes and/or microbial products, designated as microbe- or pathogen-associated molecular patterns (MAMPs/PAMPs) such as lipopolysaccharide (LPS) or peptidoglycans, and DAMPs (e.g. released from damaged enterocytes), entering the systemic circulation, where they activate different cell signaling pathways inducing a systemic gut dysbiosis-related inflammatory response [73, 74]. This gut dysbiosis-related inflammation might be a central link between the intestinal microbiota and the development of cardiometabolic diseases such as atherosclerosis [12, 73, 74]. Furthermore microbial derived metabolites such as short-chain fatty acids, primary and secondary bile acids or trimethylamine N-oxide (TMAO) and the corresponding signaling pathways have a major impact on the development of cardiovascular diseases [73]. In this context the recently described association of high protein intake with histological disease activity in NAFLD patients gains importance as a reduction in red meat consumption may offer additional health benefits for patients with NAFLD [77].

## Impact of NAFLD on cardiovascular morbidity and mortality

Liver disease in general contributes significantly to mortality in both heart failure with reduced ejection fraction (HFrEF) as well as preserved ejection fraction (HFpEF) [78]. While some cohort studies indicated an increased cardiovascular mortality in NAFLD patients [79–84], this association could not be confirmed in others [22, 85]. In most studies on this topic, associations between NAFLD and CVD were independent of established cardiovascular risk factors, such as age, sex, body mass index, waist circumference, smoking status, hypertension or dyslipidemia [27, 81, 86–88]. A recent meta-analysis of 16 observational studies (9 prospective cohort studies, 7 retrospective cohort studies) by Targher et al. including 34,043 patients with a median 7-year follow-up reported that patients with NAFLD had a 64% higher risk of developing fatal or non-fatal cardiovascular events as compared to patients without NAFLD [odds ratio (OR) 1.64; 95% confidence interval (CI) 1.26–2.13] [86]. Non-fatal events were defined as the occurrence of myocardial infarction, stroke or angina pectoris and the need for coronary revascularization. Six of the included studies indicated that particularly patients with *severe NAFLD* (defined as the presence of steatosis on imaging plus either elevated serum gamma-glutamyl-transferase concentrations, high NAFLD fibrosis score (NFS), high hepatic FDG uptake on PET-CT or advanced fibrosis stage on liver histology) were at higher risk for cardiovascular events (OR 1.94; 95% CI 1.17–3.21). These patients also had an increased cardiovascular mortality compared to patients without NAFLD (OR 3.28; 95% CI 2.26–4.77).

In contrast to the aforementioned findings, a meta-analysis of 34 studies (13 prospective cohort studies, 21 cross-sectional studies) with 164,494 participants by Wu et al. was unable to confirm a correlation between the presence of NAFLD and increased cardiovascular mortality [89]. Within their comprehensive meta-analysis, subgroup analyses of five and three suitable cohort studies, respectively, did not indicate any significant association with either overall mortality or cardiovascular mortality in both NAFL and NASH patients compared to those without NAFLD. These results, which substantially differ from those of Targher et al., can be attributed to a great heterogeneity between the patient populations of the studies included, with varying frequencies of cardiovascular comorbidities (diabetes, obesity) among the study participants, the use of different diagnostic criteria for NAFL and NASH and variable methodological quality.

However, in consideration of all studies, the meta-analysis of Wu et al. confirmed that NAFLD was associated with an increased risk for incident CVD (HR 1.37; 95% CI 1.10–1.72) and that NAFLD patients were more likely to develop coronary heart disease (HR 2.31; 95% CI 1.46–3.65) and hypertension (HR 1.16; 95% CI 1.06–1.27). In addition, it could be affirmed that the severity of NAFLD was a major determinant and that in particular the presence of NASH increases the risk of CVD (HR 2.97; 95% AI 1.03–8.52).

Disturbances in glucose metabolism and insulin resistance are key determinants in the pathogenesis of cardiovascular disease and strongly involved in the development and progression of NAFLD [83]. In this context, a recently published meta-analysis of 11 studies with 8346 patients showed that NAFLD patients with concomitant T2DM had a twofold increased risk for the manifestation of CVD when compared to patients without NAFLD (OR 2.20; 95% CI 1.67–2.90) [90].

In summary, current evidence shows that NAFLD is associated with an increased risk for the manifestation of CVD and cardiovascular events. Patients with NASH and/or advanced fibrosis (F3–F4) as well as NAFLD patients with concomitant T2DM can be identified as being part of a special risk group [27, 83, 91]. While NAFLD is associated with an increased all-cause mortality, an association with CVD mortality appears to be stage dependent. Isolated hepatic steatosis without further histological characteristics does not seem to influence cardiovascular mortality substantially. The increased all-cause mortality in the NAFLD population may primarily be driven by additional (systemic) inflammation in early disease stages and by liver-related complications in advanced fibrosis stages [18, 84, 92].

However, when evaluating the cardiovascular risk of NAFLD patients, it should always be kept in mind that NAFLD is the hepatic manifestation of the metabolic syndrome, which implies that these patients often have additional cardiometabolic risk factors. While simple steatosis alone confers less cardiovascular risk than NASH, the individual overall cardiovascular risk ultimately results from the combination of NAFLD stage and further cardiometabolic risk factors.

## Cardiovascular comorbidities in NAFLD patients

### NAFLD and arterial hypertension

Arterial hypertension is the most common modifiable risk factor for CVD. According to current World Health Organization (WHO) estimates, approximately 54% of all strokes and 47% of all cases of ischemic heart disease are a direct consequence of high blood pressure [93, 94]. In addition, arterial hypertension increases the risk for the development and progression of heart failure, peripheral arterial occlusive disease and cardiac arrhythmias, especially atrial fibrillation [94, 95].

Among NAFLD patients, the prevalence of arterial hypertension varies from 40-70% and emerging evidence has shown that NAFLD is strongly associated with an increased risk of incident prehypertension (i.e. systolic blood pressure: 120–139 mmHg, diastolic blood pressure: 80–89 mmHg) and hypertension [96, 97].

A 2–3 fold increase in the incidence of arterial hypertension was demonstrated in prospective epidemiological studies in France and Germany over observation periods of 9 and 5 years, respectively [98, 99]. Among the hypertensive or normotensive participants of the OPERA study in Finland, the 24-hour, daytime and night-time mean values of systolic or diastolic blood pressure were significantly higher among individuals with hepatic steatosis on ultrasound compared to those without NAFLD while the association with a non-dipping status has shown only a trend (30.9% vs. 24.6; *p *= 0.057) [100]. Ultrasonography revealed fatty liver more frequently when non-dipping or reverse dipping was found in 24-h ambulatory blood pressure monitoring in a group of hypertensive patients and baroreceptor sensitivity was reduced with increased blood pressure variability among NAFLD patients [101, 102].

### NAFLD and coronary artery disease

As already mentioned, the perivascular adipose tissue that surrounds the coronary arteries, promotes the expression of a proinflammatory phenotype and the accumulation of epicardial adipose tissue is closely associated with the presence, severity, and progression of coronary artery disease independently from visceral obesity [103–110]. This illuminates why NAFLD is known to be associated with various markers of subclinical atherosclerosis including coronary artery calcification and increased coronary-artery calcium score, independently from traditional CVD risk factors and metabolic syndrome features [27, 87, 111]. These markers can be used for early detection of coronary atherosclerosis and correlate with the risk for adverse cardiovascular events [27, 111–113].

In addition to subclinical atherosclerosis, NAFLD patients are also at higher risk of clinically manifesting atherosclerosis. In a meta-analysis of six studies with 25,837 patients, Mahfood Haddad et al. reported that NAFLD patients had a significantly higher risk of clinical coronary artery disease when compared to individuals without NAFLD (RR 2.26; 95% CI 1.04–4.92; *p *< 0.001) [114]. Nonalcoholic hepatic steatosis was found to be an independent predictor of high-risk coronary artery plaques that could at least in part explain the increased risk for coronary events [115]. If a vulnerable atherosclerotic plaque erodes or ruptures and an acute coronary syndrome occurs, the presence of NAFLD is associated with an unfavorable clinical outcome. In a study of 360 patients with ST segment elevation myocardial infarction (STEMI), Keskin et al. observed higher in-hospital and 3-year mortality rates in NAFLD patients than in controls [116].

### NAFLD and cardiac arrhythmias

Growing evidence also suggests, that NAFLD is associated with an increased risk of cardiac arrhythmias such as atrial fibrillation and ventricular arrhythmias [27, 112, 117–120]. As NAFLD is characterized by low-grade inflammation with increased production of proinflammatory mediators, cytokines either from the systemic circulation or locally produced within the pericardial adipose tissue, it may affect the myocardium by modulating specific ion channels resulting in the prolongation of action potential duration, which in turn increases QTc duration [121]. In addition, insulin resistance, the core feature of the metabolic syndrome, may lead to decreased potassium, affecting the prolongation of ventricular repolarization [122]. In a prospective cohort study of 400 patients with T2DM, Targher et al. observed a significant higher incidence of atrial fibrillation during a 10-year follow-up period in diabetic patients with concomitant NAFLD, as compared with diabetic subjects without NAFLD (OR 4.49; 95% CI 1.6–12.9; *p *< 0.005) [119]. These findings were confirmed by a Finnish prospective cohort study of 958 subjects by Käräjämäk et al. [120], which also demonstrated that NAFLD was a predictor for the occurrence of atrial fibrillation, even independent of the presence of T2DM.

However, there are also studies that did not confirm an association between the incidence and prevalence of NAFLD and atrial fibrillation, which can be attributed to different NAFLD definitions and differences in the prevalence of other risk factors (e.g. age and especially hypertension) among the study participants [123].

In a retrospective analysis of 238 NAFLD patients with 24-h Holter monitoring, it has also been shown that NAFLD is associated with an increased risk of prevalent ventricular arrhythmias including episodes of non-sustained ventricular tachycardia and premature ventricular complexes, independent of multiple CVD risk factors and comorbidities such as smoking, hypertension, or ischemic heart disease [118].

The increased risk of cardiac arrhythmias, especially ventricular tachyarrhythmias, could further contribute to the increased risk of CVD morbidity and mortality among NAFLD patients [124].

### NAFLD and structural heart disease

In addition to functional cardiac alterations, structural heart diseases can also be frequently observed among NAFLD patients. In clinical trials, a large number of NAFLD patients revealed myocardial remodeling, especially of the left ventricular myocardium, which is often accompanied by left ventricular systolic and/or diastolic dysfunction [124]. These structural disorders, which in turn represent another risk factor for cardiac arrhythmias, can be attributed to alterations of myocardial energy metabolism and myocardial perfusion, that can also be noted in NAFLD patients [27, 125–127].

Moreover, several trials have suggested that NAFLD is associated with the presence of aortic-valve sclerosis and mitral annulus calcification, which may favor the development of functionally relevant valve diseases in this population [128, 129]. Again, particularly NAFLD patients with concomitant T2DM seem to be at risk [129].

In summary, there is accumulating evidence that metabolic dysregulation is the common denominator that can explain the observed association of NAFLD with hypertension, coronary artery disease, cardiac arrhythmias, and structural heart disease. It stands to reason that a group of experts recently suggested metabolic (dysfunction) associated fatty liver disease “MAFLD” as a more appropriate overarching term better reflecting the pathogenesis of NAFLD [130].

### Management

The strong association between CVD and NAFLD underlines the need for early identification and adequate treatment of cardiometabolic risk factors in this population [2, 5, 8].

In this regard, international guidelines recommend that all NAFLD patients should be regularly screened for the presence of further cardiometabolic risk factors such as overweight/obesity, T2DM, dyslipidemia, and hypertension [5, 8]. In addition, all NAFLD patients with elevated biochemistry derived fibrosis scores (e.g. NFS ≥ 1.455; Fibrosis [FIB]-4-Score ≥ 1.3) should receive non-invasive measurement of liver fibrosis by transient elastography, since significant fibrosis is considered to be an important determinant of mortality from liver disease and CVD in NAFLD patients [24, 84, 91].

**Fig. 2:**
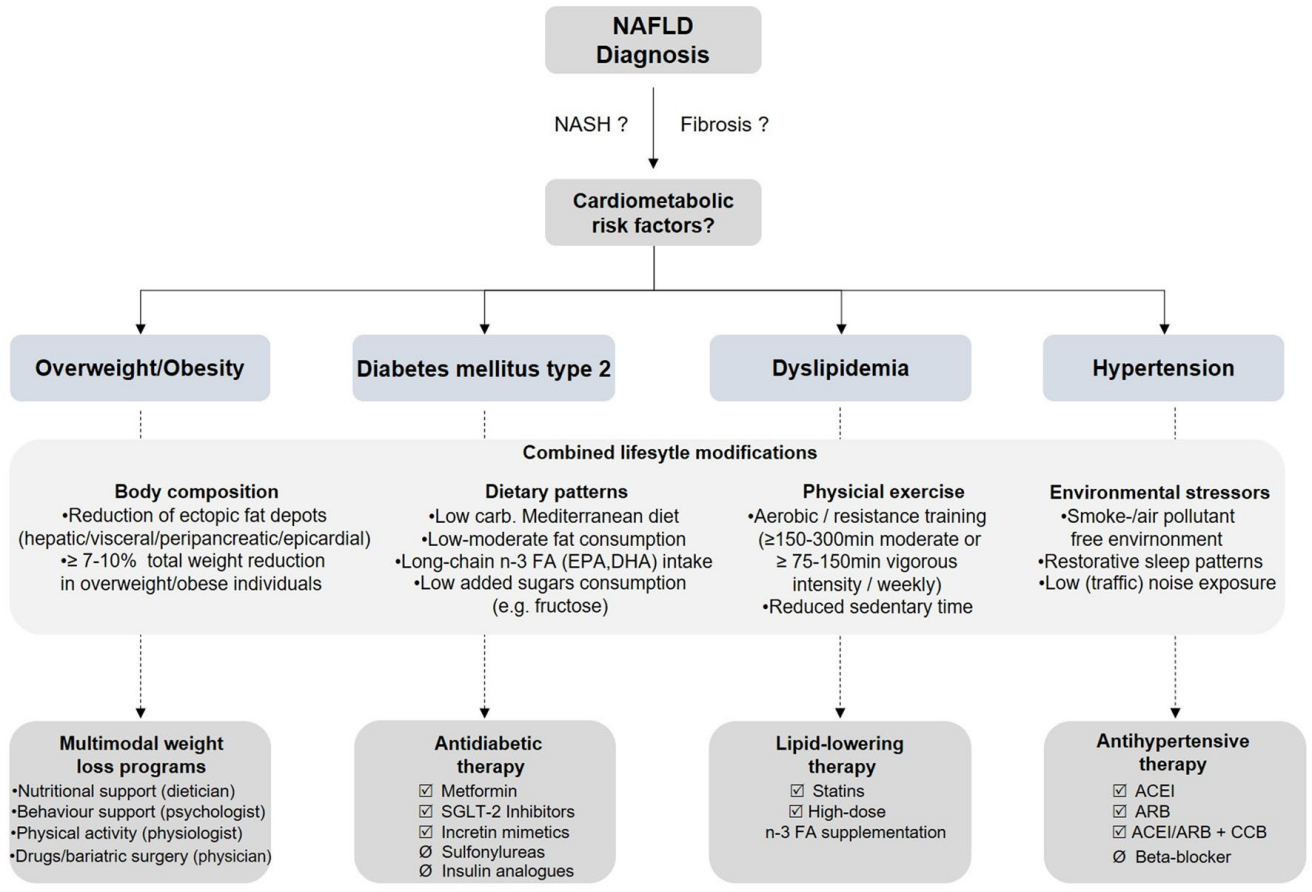
A proposed algorithm for management of cardiometabolic risk factors in NAFLD patients (modified from: [28, 29]). *NAFLD* non-alcoholic fatty liver disease, *carb*. carbohydrates, *n*-*3 FA* omega-3 fatty acids, *EPA* eicosapentaenoic acid, *DHA* docosahexaenoic acid, *min* minute, *SGLT2* sodium-glucose linked transporter 2, *ACEI* angiotensin-converting-enzyme inhibitor, *ARB* angiotensin II receptor blocker, *CCB* calcium-channel blocker

As there are no specific approved pharmacological therapies for NAFLD, an optimal management of metabolic risk factors is essential to reduce cardiovascular risk (Fig. 2).

### Non-pharmacological interventions

Lifestyle modification including dietary change, physical activity and weight loss interventions are the first-line treatment for NAFLD. In this context, pragmatic approaches combining dietary restriction and a progressive increase in aerobic or resistance training are preferable and should be individually tailored [5]. With regard to the different approaches, however, it should be considered that different lifestyle modifications variously affect specific body fat adipose depots/deposits, which is particularly relevant regarding cardiovascular comorbidities.

#### Body weight loss

In particular, weight loss is of great importance in overweight or obese NAFLD patients and can be achieved by caloric restriction, bariatric surgery, or intensive exercise [131]. While a total bodyweight loss of about 5% is already associated with a substantial reduction in liver fat content of about 30% and an improvement of metabolic abnormalities, a weight loss of at least 7–10% might be needed to substantially reduce hepatocyte inflammation, and a total weight loss of at least 10% is necessary for prognostically relevant fibrosis regression [91, 132, 133].

However, weight loss alone is insufficient to reduce the cardiovascular risk of NAFLD patients and must always be accompanied by further lifestyle modifications, including dietary and exercise interventions targeting changes in fat distribution of both adipose tissue depots (e.g. abdominal, pericardial, and renal sinus) and ectopic fat deposits (e.g. hepatic, intermuscular, and pancreatic) which are each characterized by distinct cardiometabolic properties, beyond total body fat content [32]. It is furthermore worth noting in this context that previous studies have shown that weight reduction is not obligatory for decreasing hepatic fat content or to restore normal liver function [13].

#### Physical exercise

Overall, it can be stated that any engagement in physical activity or increase over previous levels is better than continuing sedentary behaviour. In NAFLD patients, physical activity follows a dose–effect relationship and high-intensity exercise (e.g. running) rather than moderate-intensity exercise (e.g. brisk walking) shows benefits, including for NASH and fibrosis [134–136]. In this regard both aerobic exercise and resistance training effectively reduce liver fat and the choice of training should be tailored based on patients’ preferences [5, 136]. In terms of improving overall cardiometabolic health, particularly resistance training inherits beneficial effects, since it affects body composition in a way that promotes an overall anti-inflammatory and anti-atherogenic state [12, 137]. Besides positive effects on the liver fat content [138, 139], moderate physical activity, especially combined with dietary intervention, also has a great effect on decreasing visceral adipose tissue, interestingly, independent of weight loss [32]. This is very important, since reductions in both visceral adipose tissue and hepatic fat accumulation are thought to mediate beneficial cardiometabolic outcomes [140].

However, while recent data indicated an improvement in hepatic steatosis with exercise, large randomized controlled trials assessing the effect of exercise on histopathology in NASH are lacking. Current European clinical practice guidelines for NAFLD recommend 150–200 min/week of moderate intensity aerobic physical activities, such as brisk walking or stationery cycling in 3–5 sessions for NAFLD patients, as well as resistance training, that is also effective and promotes musculoskeletal fitness, with effects on concomitant metabolic risk factors [5]. This is in line with general physical exercise recommendations for improved cardiometabolic health and reduced cardiovascular morbidity in adults, namely 150–300 min of moderate-intensity or 75–150 min of vigorous-intensity activity weekly, supplemented by two sessions of muscle-building exercise and less sedentary time [141, 142].

#### Dietary changes

A high caloric diet, excess of (saturated) fat, refined carbohydrates, sugar-sweetened beverages, as well as a high fructose intake have all been associated with weight gain, obesity and NAFLD [5, 143]. Especially a high consumption of dietary fructose (e.g. by increasing use of high-fructose corn syrup in beverages) has adverse effects on metabolism, because it induces a (chronic) overproduction of intrahepatic trioses-phosphate products leading to the development of hepatic insulin resistance, intrahepatic fat accumulation via stimulated DNL and the development or aggravation of an atherogenic lipid profile [144, 145]. An isocaloric low-carbohydrate diet with < 30 g/day can lead to rapid and dramatic reductions of liver fat with marked decreases in hepatic DNL and plasma VLDL concentrations, however, the effect was no longer discernible 1–3 months later when returning to the usual pre-intervention diet [146]. Patients with NAFLD should restrict foods high in added fructose, saturated fats, simple carbohydrates and sugar containing drinks while substituting these foods with a low-to-moderate fat and moderate-to-high carbohydrate diet in addition to increasing fruits and vegetables intake [5, 147]. The macronutrient composition should be adjusted according to a Mediterranean diet, which is rich in monounsaturated fatty acids and/or diets rich in long-chain *n*-3 fatty acids eicosapentaenoic acid (EPA) and docosahexaenoic acid (DHA) which differ in their effects on cell membrane structure, rates of lipid oxidation, inflammation, and endothelial function [148, 149]. Supplementation with daily doses of 0.83 to 9.0 g resulted in beneficial changes in liver fat, plasma triglyceride and gamma-glutamyl-transferase (GGT) levels in patients with NAFLD while alanine aminotransferase (ALT) and aspartate aminotransferase (AST) remained unchanged [150]. Thus far, outcome trials have shown no cardiovascular benefit using combinations of EPA/DHA, however, icosapent-ethyl, a highly purified ethyl ester of EPA, provided large relative and absolute reductions in cardiovascular death and ischemic events in patients with established cardiovascular disease or diabetes mellitus and well-controlled LDL levels while treated with statins [151].

In a study by Ryan et al., adherence to a Mediterranean diet for six weeks was associated with significant improvements of hepatic steatosis in magnet resonance imaging when compared to a high-fat and low-carbohydrate diet, although mean weight loss was not different between these two diets [152]. Similar effects were observed in the recently published CENTRAL study. In this prospective randomized long-term intervention trial with 278 participants, Gepner et al. recently demonstrated, that a Mediterranean/low-carbohydrate diet induced a greater decrease in hepatic fat content and greater improvements in cardiometabolic risk parameters (e.g. cholesterol/HDL ratio) than a low-fat diet, conferring beneficial health effects that were beyond the favorable effects of visceral fat loss [140].

In addition to a reduction of intrahepatic fat Mediterranean diet combined with low carbohydrate consumption is also able to mobilize further atherogenic and diabetogenic ectopic fat depots, such as intrapericardial, pancreatic, or subcutaneous fat [32, 140]. In a recent study by Tsaban et al. the same diet strategy was associated with decreased intrapericardial fat volume and was independently associated with an improved lipid profile marked by a decrease in triglycerides and the ratio of triglycerides to HDL cholesterol [60].

However, focusing the different effects of lifestyle interventions on different fat depots or deposits, it should be noted that they are not all associated with the same characteristics and that they differ in terms of function and associated cardiovascular risk. While visceral, liver and pancreatic fat accumulation is associated with metabolic complications and intrapericardial fat is linked to coronary artery disease, subcutaneous adipose tissue differ significantly in terms of metabolic effects and may be considered as a largely neutral or even protective fat depot [12, 32, 153].

In view of the fact that a significant reduction in liver fat associated with improvements in lipid and glucose metabolism can only be achieved by specific dietary interventions, an integrative nutritional therapeutic concept was recently formulated that combines the most effective nutrition approaches termed “liver-fasting“ [13]. This concept involves the temporary use of low-calorie meal replacement with a specific high-protein (dairy/whey-protein), high-soluble fiber, and low-carbohydrate formula, followed by stepwise food reintroduction that implements a Mediterranean style low-carb diet for basic nutrition. This special diet strategy has been shown to significantly improve both liver fat content and the blood pressure status of patients, making it a promising therapeutic option in the context of NAFLD and CVD [13, 154].

However, it is not only important ‘what‘we eat, but also ‘when’ and ‘how often’ [155]. Recent data suggest that eating before bedtime and consuming most of the calories at dinner time is associated with a higher risk of NAFLD, whereas shifting most of the daily calorie intake to the morning or the implementation of temporary fasting episodes seems to be beneficial for weight loss and CVD health [155]. Intermittent fasting with energy restriction for about 10-16 h is promising approach in this context, as it has been shown to mediate robust disease modifying effects on chronic metabolic disorders such as obesity, T2DM and CVD [156]. In the liver, prolonged energy restriction results in a depletion of glycogen storage that subsequently triggers a metabolic switch towards the use of fatty acids and ketone bodies as energy sources [156]. This metabolic switch is accompanied by an adaptive cellular response that improves glucose metabolism, stress resistance against oxidative and metabolic stress and suppresses systemic inflammation. While growing evidence indicates that intermittent fasting is associated with numerous cardiovascular benefits, including reduced blood pressure, lipid levels, and reduced inflammatory marker, data on the effects of intermittent fasting in large cohorts of NAFLD patients are sparse [155, 156]. However, data from rodent studies demonstrated that intermittent fasting has positive effects on the reduction of hepatic steatosis and inflammation [157].

In summary, weight loss in NAFLD patients is important but insufficient as a sole measure and should always be accompanied by dietary and exercise interventions that beneficially affect fat depots linked to obesity-associated cardiometabolic morbidity.

Additional lifestyle modifying interventions, which are also of enormous relevance in the prevention of cardiometabolic diseases, include e.g. restorative sleep patterns as well as learning strategies to mitigate the physiologic response to distress [148]. This is due to the fact, that accumulating evidence suggests, that chronic exposure to stressors such as nocturnal quantitative and qualitative sleep deprivation, and psychosocial stress such as loneliness or depression should be recognized as independent risk factors for the development of metabolic disorders and cardiovascular diseases [148, 158–162]. These disturbances are closely related to sympathoadrenergic activation, chronic upregulation of the hypothalamic–pituitary– adrenal axis and cerebral and systemic chronic low grade inflammation with downstream effects on endocrine signaling and thus reveal partial overlaps with pathophysiological mechanisms that are relevant in the development of cardiometabolic diseases such as atherosclerosis, hypertension or diabetes [148, 158].

Besides individual based approaches, public health interventions aimed at decreasing sedentary time at work and the burden of environmental stressors such as tobacco smoke in public buildings, non-physiological light exposure (i.e. blue light), ambient noise, and air pollution are also urgently required and important to make the prevention of cardiometabolic diseases even more comprehensive [31, 148].

### Pharmacological interventions

Considering pharmacological therapies for the risk group of NAFLD patients with concomitant T2DM, it needs to be considered that some glucose-lowering drugs promote adipogenesis and the accumulation of e.g. epicardial fat (e.g. sulfonylureas, insulin/insulin analogues), whereas other antidiabetic drugs (e.g. GLP-1 (glucagon-like peptide-1 receptor agonists, dipeptidyl peptidase-4 inhibitors) reduce the accumulation of ectopic fat but do not reduce inflammation. In contrast, both metformin and SGLT2 (sodium glucose-linked transporter 2) inhibitors reduce the quantity of ectopic fat and ameliorate its inflammation as well as its secretion of adipokines [59]. Especially, the novel anti-diabetic drugs such as GLP-1 analogues and SGLT2 inhibitors represent promising options, as they can reduce the risk for adverse cardiovascular events in diabetic patients and show beneficial effects on the liver [91, 163, 164].

It is also noteworthy, that SGLT2 inhibitors induce a transcriptional paradigm that closely mimics the cellular response to starvation [165]. Via activation of the SIRT1/AMPK signaling pathway and suppression of the Akt/mTOR signaling pathway, SGLT2 inhibitors induce a variety of beneficial effects: reduction in oxidative stress, normalization of mitochrondrial structure and function, suppression of inflammation, minimization of coronary microvascular injury, enhancement of contractile performance, and attenuation of the development of cardiomyopathy. This is paralleled by an amelioration of glomerular and tubular inflammation and injury alleviating the development of nephropathy [165]. This particularly favourable influence the pathogenesis of cardiac and renal disease, will probably lead to an increased use of these substances in the future, even in non-diabetic patients.

The main therapeutic aim in the treatment of coincident dyslipidemia is LDL-cholesterol lowering, preferably with statins, which has been proven to be safe in NAFLD [166]. Statins are characterized by anti-inflammatory, anti-oxidative, anti-fibrotic and plaque-stabilizing effects, whereby they may improve vascular and hepatic function among NAFLD patients and reduce cardiovascular risk.

In NAFLD patients with hypertension angiotensin-converting enzyme inhibitors and angiotensin receptor blockers have been noted as a promising medication, since the renin–angiotensin–aldosterone system is involved in the pathogenesis of both NAFLD and cardiovascular pathologies. Although several studies indicated beneficial effects of renin-angiotensin system blockers on histological fibrosis progression in NAFLD, current evidence is insufficient to recommend renin-angiotensin system blockers only for the purpose of managing fibrosis in NAFLD patients [167].

At the interface of CVD and NAFLD, acetylsalicylic acid *(*ASA) is another promising approach. ASA is indicated in secondary prevention in all patients with established CVD including coronary heart disease, previous myocardial infarction, previous stroke, and peripheral arterial disease. Interestingly, recent studies suggest that ASA may also have antifibrotic effects in NAFLD. In a prospective cohort study of 361 patients with biopsy-proven NAFLD, daily ASA use was associated with less severe histologic features of NAFLD and NASH, and lower risk for progression to advanced fibrosis with time [168]. The greatest benefit was seen in patients with at least 4 years or more of ASA use.

Although the underlying hepatoprotective effects of ASA to prevent fibrogenesis in NAFLD need further clarification, it is an interesting approach, which could also have a beneficial effect on the cardiovascular risk of a subgroup of patients.

## Conclusion

In summary, the individual cardiovascular risk is already increased in early NAFLD stages. Patients with NASH and/or advanced fibrosis (F3-F4) as well as NAFLD patients with concomitant T2DM can be identified as special risk groups, which have the highest risk for adverse cardiovascular events and mortality. The diagnosis of NAFLD deserves a thoughtful cardiovascular risk assessment and evaluation for subclinical atherosclerosis. This has the potential to enable improved identification of high-risk patients who are candidates for therapeutic interventions in order to address the unacceptable global disease burden attributable to the intertwined pandemic of metabolic and cardiovascular diseases. Lifestyle modifications including weight loss, increased physical activity and adjusted nutrition, e.g. Mediterranean/low-carbohydrate diet, are the basis of NAFLD prevention and treatment. While there is currently no approved specific drug treatment for the liver itself, concomitant cardiovascular risk factors should be targeted through the use of statins, antihypertensive drugs, preferably ACE inhibitors and angiotensin-receptor blockers, aspirin and insulin-sensitizing agents like glucagon-like peptide-1 analogues or metformin and probably SGLT2 inhibitors.

## References

[1] Diehl A, Day C (2017). Cause, pathogenesis, and treatment of nonalcoholic steatohepatitis. N Engl J Med.

[2] Roeb E, Steffen H, Bantel H (2015). S2k guideline non-alcoholic fatty liver disease. Z Gastroenterol.

[3] McPherson S, Hardy T, Henderson E (2015). Evidence of NAFLD progression from steatosis to fibrosing-steatohepatitis using paired biopsies: implications for prognosis and clinical management. J Hepatol.

[4] Medina-Santillán R, López-Velázquez J, Chávez-Tapia N (2013). Hepatic manifestations of metabolic syndrome. Diabetes Metab Res Rev.

[5] Marchesini G, Day C, Dufour J (2016). EASL-EASD-EASO clinical practice guidelines for the management of non-alcoholic fatty liver disease. J Hepatol.

[6] Wong V, Chan W, Chitturi S (2018). Asia-pacific working party on non-alcoholic fatty liver disease guidelines 2017-part 1: definition, risk factors and assessment. J Gastroenterol Hepatol.

[7] Brunt E, Neuschwander-Tetri B, Oliver D (2004). Nonalcoholic steatohepatitis: histologic features and clinical correlations with 30 blinded biopsy specimens. Hum Pathol.

[8] Chalasani N, Younossi Z, Lavine J (2018). The diagnosis and management of nonalcoholic fatty liver disease: practice guidance from the American Association for the study of liver diseases. Hepatology.

[9] Ratziu V, Sanyal A, Harrison S (2020). Cenicriviroc treatment for adults with nonalcoholic steatohepatitis and fibrosis: final analysis of the phase 2b CENTAUR study. Hepatology.

[10] Calzadilla Bertot L, Adams L (2016). The natural course of non-alcoholic fatty liver disease. Int J Mol Sci.

[11] Loomba R, Adams L (2019). The 20% rule of NASH progression: the natural history of advanced fibrosis and cirrhosis caused by NASH. Hepatology.

[12] Lechner K, McKenzie A, Kränkel N (2020). High-risk atherosclerosis and metabolic phenotype: the roles of ectopic adiposity, atherogenic dyslipidemia, and inflammation. Metab Syndr Relat Disord.

[13] Worm N (2020). Beyond body weight-loss: dietary strategies targeting intrahepatic fat in NAFLD. Nutrients.

[14] Younossi Z (2019). Non-alcoholic fatty liver disease—a global public health perspective. J Hepatol.

[15] Younossi Z, Tacke F, Arrese M (2019). Global perspectives on nonalcoholic fatty liver disease and nonalcoholic steatohepatitis. Hepatology.

[16] Younossi Z, Koenig A, Abdelatif D (2016). Global epidemiology of nonalcoholic fatty liver disease-meta-analytic assessment of prevalence, incidence, and outcomes. Hepatology.

[17] Dulai P, Singh S, Patel J (2017). Increased risk of mortality by fibrosis stage in nonalcoholic fatty liver disease: systematic review and meta-analysis. Hepatology.

[18] Vilar-Gomez E, Calzadilla-Bertot L, Wai-Sun Wong V (2018). Fibrosis severity as a determinant of cause-specific mortality in patients with advanced nonalcoholic fatty liver disease: a multi-national cohort study. Gastroenterology.

[19] Haflidadottir S, Jonasson J, Norland H (2014). Long-term follow-up and liver-related death rate in patients with non-alcoholic and alcoholic related fatty liver disease. BMC Gastroenterol.

[20] Angulo P, Kleiner D, Dam-Larsen S (2015). Liver fibrosis, but no other histologic features, is associated with long-term outcomes of patients with nonalcoholic fatty liver disease. Gastroenterology.

[21] Nasr P, Ignatova S, Kechagias S, Ekstedt M (2017). Natural history of nonalcoholic fatty liver disease: a prospective follow-up study with serial biopsies. Hepatol Commun.

[22] Kim D, Kim W, Kim H, Therneau T (2013). Association between noninvasive fibrosis markers and mortality among adults with nonalcoholic fatty liver disease in the United States. Hepatology.

[23] Ekstedt M, Franzén L, Mathiesen U (2006). Long-term follow-up of patients with nafld and elevated liver enzymes. Hepatology.

[24] Hagström H, Nasr P, Ekstedt M (2017). Fibrosis stage but not nash predicts mortality and time to development of severe liver disease in biopsy-proven NAFLD. J Hepatol.

[25] Estes C, Anstee Q, Arias-Loste M (2018). Modeling NAFLD disease burden in China, France, Germany, Italy, Japan, Spain, United Kingdom, and United States for the Period 2016–2030. J Hepatol.

[26] Janssen A, Grobbee D, Dendale P (2020). Non-alcoholic fatty liver disease, a new and growing risk indicator for cardiovascular disease. Eur J Prev Cardiol.

[27] Adams L, Anstee Q, Tilg H, Targher G (2017). Non-alcoholic fatty liver disease and its relationship with cardiovascular disease and other extrahepatic diseases. Gut.

[28] Stahl E, Dhindsa D, Lee S (2019). Nonalcoholic fatty liver disease and the heart: JACC state of the art review. J Am Coll Cardiol.

[29] Francque S, van der Graaff D, Kwanten W (2016). Non-alcoholic fatty liver disease and cardiovascular risk: pathophysiological mechanisms and implications. J Hepatol.

[30] Cusi K (2012). Role of obesity and lipotoxicity in the development of nonalcoholic steatohepatitis: pathophysiology and clinical implications. Gastroenterology.

[31] Lechner K, Lorenz E, Drezner J (2020). The “heart” of preventive cardiology: lifestyle medicine for the treatment of cardiometabolic diseases. Eur J Prev Cardiol.

[32] Gepner Y, Shelef I, Schwarzfuchs D (2018). Effect of distinct lifestyle interventions on mobilization of fat storage pools: CENTRAL magnetic resonance imaging randomized controlled trial. Circulation.

[33] Ipsen D, Lykkesfeldt J, Tveden-Nyborg P (2018). Molecular mechanisms of hepatic lipid accumulation in non-alcoholic fatty liver disease. Cell Mol Life Sci.

[34] Adiels M, Olofsson S, Taskinen M, Borén J (2008). Overproduction of very low-density lipoproteins is the hallmark of the dyslipidemia in the metabolic syndrome. Arter Thromb Vasc Biol.

[35] Borén J, Chapman M, Krauss R (2020). Low-density lipoproteins cause atherosclerotic cardiovascular disease: pathophysiological, genetic, and therapeutic insights: a consensus statement from the European Atherosclerosis Society Consensus Panel. Eur Hear J.

[36] Zewinger S, Reiser J, Jankowski V (2020). Apolipoprotein C3 induces inflammation and organ damage by alternative inflammasome activation. Nat Immunol.

[37] Goulopoulou S, McCarthy C, Webb R (2016). Toll-like receptors in the vascular system: sensing the dangers within. Pharmacol Rev.

[38] Libby P, Everett B (2019). Novel antiatherosclerotic therapies. Arter Thromb Vasc Biol.

[39] Al-Mrabeh A, Zhyzhneuskaya S, Peters C (2020). Hepatic lipoprotein export and remission of human type 2 diabetes after weight loss. Cell Metab.

[40] Hwang DH, Kim JALJ (2016). Mechanisms for the activation of toll-like receptor 2/4 by saturated fatty acids and inhibition by docosahexaenoic acid. Eur J Pharmacol.

[41] Lai H, de Oliveira Otto M, Lee Y (2019). Serial plasma phospholipid fatty acids in the de novo lipogenesis pathway and total mortality, cause-specific mortality, and cardiovascular diseases in the cardiovascular health study. J Am Heart Assoc.

[42] Volk B, Kunces L, Freidenreich D (2014). Effects of step-wise increases in dietary carbohydrate on circulating saturated fatty acids and palmitoleic acid in adults with metabolic syndrome. PLoS ONE.

[43] Lee J, Lambert J, Hovhannisyan Y (2015). Palmitoleic acid is elevated in fatty liver disease and reflects hepatic lipogenesis. Am J Clin Nutr.

[44] Shulman G (2014). Ectopic fat in insulin resistance, dyslipidemia, and cardiometabolic disease. N Engl J Med.

[45] Petersen M, Shulman G (2018). Mechanisms of insulin action and insulin resistance. Physiol Rev.

[46] White M, Shaw J, Taylor R (2016). Type 2 diabetes: the pathologic basis of reversible β-cell dysfunction. Diabetes Care.

[47] Low Wang C, Hess C, Hiatt W, Goldfine A (2016). Clinical update: cardiovascular disease in diabetes mellitus: atherosclerotic cardiovascular disease and heart failure in type 2 diabetes mellitus—mechanisms, management, and clinical considerations. Circulation.

[48] Agarwal S, Norby F, Whitsel E (2017). Cardiac autonomic dysfunction and incidence of atrial fibrillation in a large population-based cohort. J Am Coll Cardiol.

[49] Anand S, Yi Q, Gerstein H (2003). Relationship of metabolic syndrome and fibrinolytic dysfunction to cardiovascular disease. Circulation.

[50] Laakso M, Kuusisto J (2014). Insulin resistance and hyperglycaemia in cardiovascular disease development. Nat Rev Endocrinol.

[51] Villanova N, Moscatiello S, Ramilli S (2005). Endothelial dysfunction and cardiovascular risk profile in nonalcoholic fatty liver disease. Hepatology.

[52] Scholz G, Hanefeld M (2016). Metabolic vascular syndrome: new insights into a multidimensional network of risk factors and diseases. Visc Med.

[53] Pastore A, Alisi A, di Giovamberardino G (2014). Plasma levels of homocysteine and cysteine increased in pediatric nafld and strongly correlated with severity of liver damage. Int J Mol Sci.

[54] de Carvalho S, Muniz M, Siqueira M (2013). Plasmatic higher levels of homocysteine in non-alcoholic fatty liver disease (NAFLD). Nutr J.

[55] Xu Y, Guan Y, Yang X (2020). Association of serum homocysteine levels with histological severity of NAFLD. J Gastrointestin Liver Dis.

[56] Tripodi A, Fracanzani A, Primignani M (2014). Procoagulant imbalance in patients with non-alcoholic fatty liver disease. J Hepatol.

[57] Coulon S, Francque S, Colle I (2012). Evaluation of inflammatory and angiogenic factors in patients with non-alcoholic fatty liver disease. Cytokine.

[58] Després J (2012). Body fat distribution and risk of cardiovascular disease: an update. Circulation.

[59] Packer M (2018). Epicardial adipose tissue may mediate deleterious effects of obesity and inflammation on the myocardium. Cardiol J Am Coll.

[60] Tsaban G, Wolak A, Avni-Hassid H (2017). Dynamics of intrapericardial and extrapericardial fat tissues during long-term, dietary-induced, moderate weight loss. Am J Clin Nutr.

[61] Gaborit B, Sengenes C, Ancel P (2017). Role of epicardial adipose tissue in health and disease: a matter of fat?. Compr Physiol.

[62] Mariani S, Fiore D, Barbaro G (2013). Association of epicardial fat thickness with the severity of obstructive sleep apnea in obese patients. Int J Cardiol.

[63] Monfort A, Inamo J, Fagour C (2019). Epicardial fat accumulation is an independent marker of impaired heart rate recovery in obese patients with obstructive sleep apnea. Clin Res Cardiol.

[64] Fracanzani A, Pisano G, Consonni D (2016). Epicardial adipose tissue (eat) thickness is associated with cardiovascular and liver damage in nonalcoholic fatty liver disease. PLoS ONE.

[65] Gruzdeva O, Uchasova E, Dyleva Y (2019). Adipocytes directly affect coronary artery disease pathogenesis via induction of adipokine and cytokine imbalances. Front Immunol.

[66] Packer M (2020). Atrial fibrillation and heart failure with preserved ejection fraction in patients with nonalcoholic fatty liver disease. Am J Med.

[67] Abul-Husn N, Cheng X, Li A (2018). A protein-truncating HSD17B13 variant and protection from chronic liver disease. N Engl J Med.

[68] Emdin C, Haas M, Khera A (2020). A missense variant in mitochondrial amidoxime reducing component 1 gene and protection against liver disease. PLoS Genet.

[69] Eslam M, Valenti L, Romeo S (2018). Genetics and epigenetics of NAFLD and NASH: clinical impact. J Hepatol.

[70] Dongiovanni P, Petta S, Maglio C (2015). Transmembrane 6 superfamily member 2 gene variant disentangles nonalcoholic steatohepatitis from cardiovascular disease. Hepatology.

[71] Lauridsen B, Stender S, Kristensen T (2018). Liver fat content, non-alcoholic fatty liver disease, and ischaemic heart disease: mendelian randomization and meta-analysis of 279 013 individuals. Eur Hear J.

[72] Liu D, Peloso G, Yu H (2017). Exome-wide association study of plasma lipids in > 300000 individuals. Nat Genet.

[73] Tang W, Bäckhed F, Landmesser U, Hazen S (2019). Intestinal microbiota in cardiovascular health and disease: JACC state-of-the-art review. J Am Coll Cardiol.

[74] Ma J, Li H (2018). The role of gut microbiota in atherosclerosis and hypertension. Front Pharmacol.

[75] Aron-Wisnewsky J, Vigliotti C, Witjes J (2020). Gut microbiota and human NAFLD: disentangling microbial signatures from metabolic disorders. Nat Rev Gastroenterol Hepatol.

[76] Demir M, Lang S, Martin A (2020). Phenotyping non-alcoholic fatty liver disease by the gut microbiota: ready for prime time?. J Gastroenterol Hepatol.

[77] Lang S, Martin A, Farowski F (2020). High protein intake is associated with histological disease activity in patients with NAFLD. Hepatol Commun.

[78] Ergatoudes C, Schaufelberger M, Andersson B (2019). Non-cardiac comorbidities and mortality in patients with heart failure with reduced vs. preserved ejection fraction: a study using the swedish heart failure registry. Clin Res Cardiol.

[79] Zhou Y, Li Y, Nie Y (2012). Natural course of nonalcoholic fatty liver disease in southern china: a prospective cohort study. J Dig Dis.

[80] Söderberg C, Stål P, Askling J (2010). Decreased survival of subjects with elevated liver function tests during a 28-year follow-up. Hepatology.

[81] Zeb I, Li D, Budoff M (2016). Nonalcoholic fatty liver disease and incident cardiac events: the multi-ethnic study of atherosclerosis. J Am Coll Cardiol.

[82] Allen A, Therneau T, Larson J (2018). Nonalcoholic fatty liver disease incidence and impact on metabolic burden and death: a 20 year-community study. Hepatology.

[83] Wong C, Lim J (2018). The association between nonalcoholic fatty liver disease and cardiovascular disease outcomes. Clin Liver Dis.

[84] Ekstedt M, Hagström H, Nasr P (2015). Fibrosis stage is the strongest predictor for disease-specific mortality in NAFLD after up to 33 years of follow-up. Hepatology.

[85] Lazo M, Hernaez R, Bonekamp S (2011). Non-alcoholic Fatty liver disease and mortality among us adults: prospective cohort study. BMJ.

[86] Targher G, Byrne C, Lonardo A (2016). Non-alcoholic fatty liver disease and risk of incident cardiovascular disease: a meta-analysis. J Hepatol.

[87] Oni E, Agatston A, Blaha M (2013). A systematic review: burden and severity of subclinical cardiovascular disease among those with nonalcoholic fatty liver; should we care?. Atherosclerosis.

[88] Mantovani A, Mingolla L, Rigolon R (2016). nonalcoholic fatty liver disease is independently associated with an increased incidence of cardiovascular disease in adult patients with type 1 diabetes. Int J Cardiol.

[89] Wu S, Wu F, Ding Y (2016). Association of non-alcoholic fatty liver disease with major adverse cardiovascular events: a systematic review and meta-analysis. Sci Rep.

[90] Zhou Y, Zhou X, Wu S (2018). Synergistic increase in cardiovascular risk in diabetes mellitus with nonalcoholic fatty liver disease: a meta-analysis. Eur J Gastroenterol Hepatol.

[91] Stefan N, Häring H, Cusi K (2019). Non-alcoholic fatty liver disease: causes, diagnosis, cardiometabolic consequences, and treatment strategies. Lancet Diabetes Endocrinol.

[92] Taylor R, Taylor R, Bayliss S (2020). Association between fibrosis stage and outcomes of patients with non-alcoholic fatty liver disease: a systematic review and meta-analysis. Gastroenteorlogy.

[93] James S, Abate D, Abate K (2018). Global, regional, and national incidence, prevalence, and years lived with disability for 354 diseases and injuries for 195 countries and territories, 1990-2017: a systematic analysis for the global burden of disease study 2017. Lancet.

[94] Jordan J, Kurschat C, Reuter H (2018). Arterial Hypertension. Dtsch Arztebl Int.

[95] Kjeldsen S (2018). Hypertension and cardiovascular risk: general aspects. Pharmacol Res.

[96] Aneni E, Oni E, Martin S (2015). Blood pressure is associated with the presence and severity of nonalcoholic fatty liver disease across the spectrum of cardiometabolic risk. J Hypertens.

[97] Ryoo J, Suh Y, Shin H (2014). Clinical association between non-alcoholic fatty liver disease and the development of hypertension. J Gastroenterol Hepatol.

[98] Bonnet F, Gastaldelli A, Pihan-Le Bars F (2017). Gamma-glutamyltransferase, fatty liver index and hepatic insulin resistance are associated with incident hypertension in two longitudinal studies. J Hypertens.

[99] Lau K, Lorbeer R, Haring R (2010). The association between fatty liver disease and blood pressure in a population-based prospective longitudinal study. J Hypertens.

[100] Vasunta R, Kesäniemi Y, Ylitalo A, Ukkola O (2012). Primary non-alcoholic fatty liver disease in hypertensive patients. J Hypertens.

[101] Latea L, Negrea S, Bolboaca S (2013). Primary non-alcoholic fatty liver disease in hypertensive patients. Aust Med J.

[102] Singh A, Kumar M, Jaryal A (2017). Diabetic status and grade of nonalcoholic fatty liver disease are associated with lower baroreceptor sensitivity in patients with nonalcoholic fatty liver disease. Eur J Gastroenterol Hepatol.

[103] Thalmann S, Meier C (2007). Local adipose tissue depots as cardiovascular risk factors. Cardiovasc Res.

[104] Mach L, Bedanova H, Soucek M (2017). Impact of cardiopulmonary bypass surgery on cytokines in epicardial adipose tissue: comparison with subcutaneous fat. Perfusion.

[105] Hirata Y, Kurobe H, Akaike M (2011). Enhanced inflammation in epicardial fat in patients with coronary artery disease. Int Heart J.

[106] Shibasaki I, Nishikimi T, Mochizuki Y (2010). Greater expression of inflammatory cytokines, adrenomedullin, and natriuretic peptide receptor-c in epicardial adipose tissue in coronary artery disease. Regul Pept.

[107] Sinha S, Thakur R, Jha M (2016). Epicardial adipose tissue thickness and its association with the presence and severity of coronary artery disease in clinical setting: a cross-sectional observational study. J Clin Med Res.

[108] Picard F, Gueret P, Laissy J (2014). Epicardial adipose tissue thickness correlates with the presence and severity of angiographic coronary artery disease in stable patients with chest pain. PLoS ONE.

[109] Yerramasu A, Dey D, Venuraju S (2012). Increased volume of epicardial fat is an independent risk factor for accelerated progression of sub-clinical coronary atherosclerosis. Atherosclerosis.

[110] Okada K, Ohshima S, Isobe S (2014). Epicardial fat volume correlates with severity of coronary artery disease in nonobese patients. J Cardiovasc Med.

[111] Jaruvongvanich V, Wirunsawanya K, Sanguankeo A, Upala S (2016). Nonalcoholic fatty liver disease is associated with coronary artery calcification: a systematic review and meta-analysis. Dig Liver Dis.

[112] Sinn D, Kang D, Chang Y (2017). Non-alcoholic fatty liver disease and progression of coronary artery calcium score: a retrospective cohort study. Gut.

[113] Chang Y, Ryu S, Sung K (2019). Alcoholic and non-alcoholic fatty liver disease and associations with coronary artery calcification: evidence from the kangbuk samsung health study. Gut.

[114] Mahfood Haddad T, Hamdeh S, Kanmanthareddy A, Alla V (2017). Nonalcoholic fatty liver disease and the risk of clinical cardiovascular events: a systematic review and meta-analysis. Diabetes Metab Syndr.

[115] Osawa K, Miyoshi T, Yamauchi K (2015). Nonalcoholic hepatic steatosis is a strong predictor of high-risk coronary-artery plaques as determined by multidetector CT. PLoS ONE.

[116] Keskin M, Hayıroğlu M, Uzun A (2017). Effect of nonalcoholic fatty liver disease on in-hospital and long-term outcomes in patients with st-segment elevation myocardial infarction. Am J Cardiol.

[117] Mantovani A (2017). Nonalcoholic fatty liver disease (NAFLD) and risk of cardiac arrhythmias: a new aspect of the liver-heart axis. J Clin Transl Hepatol.

[118] Mantovani A, Rigamonti A, Bonapace S (2016). Nonalcoholic fatty liver disease is associated with ventricular arrhythmias in patients with type 2 diabetes referred for clinically indicated 24-hour holter monitoring. Diabetes Care.

[119] Targher G, Valbusa F, Bonapace S (2013). Non-alcoholic fatty liver disease is associated with an increased incidence of atrial fibrillation in patients with type 2 diabetes. PLoS ONE.

[120] Käräjämäki A, Pätsi O, Savolainen M (2015). Non-alcoholic fatty liver disease as a predictor of atrial fibrillation in middle-aged population (opera study). PLoS ONE.

[121] Lazzerini P, Capecchi P, Laghi-Pasini F (2015). Long QT syndrome: an emerging role for inflammation and immunity. Front Cardiovasc Med.

[122] Kim H, Lee D, Lee S, Koh G (2015). A relationship between serum potassium concentration and insulin resistance in patients with type 2 diabetes mellitus. Int Urol Nephrol.

[123] Long M, Yin X, Larson M (2017). Relations of liver fat with prevalent and incident atrial fibrillation in the framingham heart study. J Am Heart Assoc.

[124] Anstee Q, Mantovani A, Tilg H, Targher G (2018). Risk of cardiomyopathy and cardiac arrhythmias in patients with nonalcoholic fatty liver disease. Nat Rev Gastroenterol Hepatol.

[125] Hallsworth K, Hollingsworth K, Thoma C (2013). Association of nonalcoholic fatty liver disease with subclinical myocardial remodeling and dysfunction: a population-based study. J Hepatol.

[126] VanWagner L, Wilcox J, Colangelo L (2015). Association of nonalcoholic fatty liver disease with subclinical myocardial remodeling and dysfunction: a population-based study. Hepatology.

[127] Trovato F, Martines G, Catalano D (2016). Echocardiography and NAFLD (non-alcoholic fatty liver disease). Int J Cardiol.

[128] Bonapace S, Valbusa F, Bertolini L (2014). Nonalcoholic fatty liver disease is associated with aortic valve sclerosis in patients with type 2 diabetes mellitus. PLoS ONE.

[129] Mantovani A, Pernigo M, Bergamini C (2015). Heart valve calcification in patients with type 2 diabetes and nonalcoholic fatty liver disease. Metabolism.

[130] Eslam M, Newsome P, Sarin S (2020). A new definition for metabolic dysfunction-associated fatty liver disease: an international expert consensus statement. J Hepatol.

[131] Koutoukidis D, Astbury N, Tudor K (2019). Association of weight loss interventions with changes in biomarkers of nonalcoholic fatty liver disease: a systematic review and meta-analysis. JAMA Intern Med.

[132] Promrat K, Kleiner D, Niemeier H (2010). Randomized controlled trial testing the effects of weight loss on nonalcoholic steatohepatitis (NASH). Hepatology.

[133] Glass L, Dickson R, Anderson J (2015). Total body weight loss of ≥ 10% is associated with improved hepatic fibrosis in patients with nonalcoholic steatohepatitis. Dig Dis Sci.

[134] Thoma C, Day C, Trenell M (2012). Lifestyle interventions for the treatment of non-alcoholic fatty liver disease in adults: a systematic review. J Hepatol.

[135] Keating S, Hackett D, George J, Johnson N (2012). Exercise and non-alcoholic fatty liver disease: a systematic review and meta-analysis. J Hepatol.

[136] Bacchi E, Negri C, Targher G (2013). Both resistance training and aerobic training reduce hepatic fat content in type 2 diabetic subjects with nonalcoholic fatty liver disease (the raed2 randomized trial). Hepatology.

[137] Fiuza-Luces C, Santos-Lozano A, Joyner M (2018). Exercise benefits in cardiovascular disease: beyond attenuation of traditional risk factors. Nat Rev Cardiol.

[138] Oh S, So R, Shida S (2017). High-intensity aerobic exercise improves both hepatic fat content and stiffness in sedentary obese men with nonalcoholic fatty liver disease. Sci Rep.

[139] Sung K, Ryu S, Lee J (2016). Effect of exercise on the development of new fatty liver and the resolution of existing fatty liver. J Hepatol.

[140] Gepner Y, Shelef I, Komy O (2019). The beneficial effects of mediterranean diet over low-fat diet may be mediated by decreasing hepatic fat content. J Hepatol.

[141] Piercy K, Troiano R, Ballard R (2018). The physical activity guidelines for americans. JAMA.

[142] Fletcher G, Landolfo C, Niebauer J (2018). Promoting physical activity and exercise: JACC health promotion series. J Am Coll Cardiol.

[143] Barrera F, George J (2014). The role of diet and nutritional intervention for the management of patients with NAFLD. Clin Liver Dis.

[144] Schwarz J, Noworolski S, Wen M (2015). Effect of a high-fructose weight-maintaining diet on lipogenesis and liver fat. J Clin Endocrinol Metab.

[145] Tappy L (2018). Fructose-containing caloric sweeteners as a cause of obesity and metabolic disorders. J Exp Biol.

[146] Mardinoglu A, Wu H, Bjornson E (2018). An integrated understanding of the rapid metabolic benefits of a carbohydrate-restricted diet on hepatic steatosis in humans. Cell Metab.

[147] Ismaiel A, Dumitraşcu D (2019). Cardiovascular risk in fatty liver disease: the liver-heart axis-literature review. Front Med.

[148] Lechner K, von Schacky C, McKenzie A (2020). Lifestyle factors and high-risk atherosclerosis: pathways and mechanisms beyond traditional risk factors. Eur J Prev Cardiol.

[149] Mason R, Libby P, Bhatt D (2020). Emerging mechanisms of cardiovascular protection for the omega-3 fatty acid eicosapentaenoic acid. Arter Thromb Vasc Biol.

[150] Lu W, Li S, Li J (2016). Effects of omega-3 fatty acid in nonalcoholic fatty liver disease: a meta-analysis. Gastroenterol Res Pr.

[151] Bhatt D, Steg P, Miller M (2019). Cardiovascular risk reduction with icosapent ethyl for hypertriglyceridemia. N Engl J Med.

[152] Ryan M, Itsiopoulos C, Thodis T (2013). The mediterranean diet improves hepatic steatosis and insulin sensitivity in individuals with non-alcoholic fatty liver disease. J Hepatol.

[153] Golan R, Shelef I, Rudich A (2012). Abdominal superficial subcutaneous fat: a putative distinct protective fat subdepot in type 2 diabetes. Diabetes Care.

[154] Schweinlin A, Ulbrich S, Stauß S (2018). Comparison of a commercially available, formula-based nutritional therapy enriched with oats fiber with a non-formula isocaloric therapy to treat non-alcoholic fatty liver disease (NAFLD)—a randomized, controlled intervention trial. Z Gastroenterol.

[155] El-Agroudy N, Kurzbach A, Rodionov R (2019). Are lifestyle therapies effective for NAFLD treatment?. Trends Endocrinol Metab.

[156] de Cabo R, Mattson MP (2019). Effects of intermittent fasting on health, aging, and disease. N Engl J Med.

[157] Marinho T, Ornellas F, Barbosa-da-Silva S (2019). Beneficial effects of intermittent fasting on steatosis and inflammation of the liver in mice fed a high-fat or a high-fructose diet. Nutrition.

[158] Münzel T, Sørensen M, Gori T (2017). Environmental stressors and cardio-metabolic disease: part i-epidemiologic evidence supporting a role for noise and air pollution and effects of mitigation strategies. Eur Heart J.

[159] Münzel T, Sørensen M, Gori T (2017). Environmental stressors and cardio-metabolic disease: part II-mechanistic insights. Eur Heart J.

[160] Koren D, Taveras E (2018). Association of sleep disturbances with obesity, insulin resistance and the metabolic syndrome. Metabolism.

[161] Vaccarino V, Badimon L, Bremner J (2020). Depression and coronary heart disease: 2018 position paper of the ESC working group on coronary pathophysiology and microcirculation. Eur Heart J.

[162] Dar T, Radfar A, Abohashem S (2019). Psychosocial stress and cardiovascular disease. Curr Treat Options Cardiovasc Med.

[163] Raj H, Durgia H, Palui R (2019). SGLT-2 inhibitors in non-alcoholic fatty liver disease patients with type 2 diabetes mellitus: a systematic review. World J Diabetes.

[164] Barb D, Portillo-Sanchez P, Cusi K (2016). Pharmacological management of nonalcoholic fatty liver disease. Metabolism.

[165] Packer M (2020). SGLT2 inhibitors produce cardiorenal benefits by promoting adaptive cellular reprogramming to induce a state of fasting mimicry: a paradigm shift in understanding their mechanism of action. Diabetes Care.

[166] Pastori D, Polimeni L, Baratta F (2015). The efficacy and safety of statins for the treatment of non-alcoholic fatty liver disease. Dig Liver Dis.

[167] Li Y, Xu H, Wu W (2018). Clinical application of angiotensin receptor blockers in patients with non-alcoholic fatty liver disease: a systematic review and meta-analysis. Oncotarget.

[168] Simon T, Henson J, Osganian S (2019). Daily aspirin use associated with reduced risk for fibrosis progression in patients with nonalcoholic fatty liver disease. Clin Gastroenterol Hepatol.

